# Microneedle-Delivered Plant-Derived Exosome-like Nanovesicles for Dermatological Diseases: A Comprehensive Review

**DOI:** 10.3390/ph19101579

**Published:** 2026-10-05

**Authors:** Letong Huang, Hailong Zhang, Jiming Han, Maoyuan Zeng, Hongqin Sun, Yi Huang, Yuxiang Li, Siyu Chen, Mingtao Ma, Tao Song, Jidong Zhang

**Affiliations:** 1 Department of Immunology, Zunyi Medical University, Zunyi 563000, China; zmuhlt0530@163.com (L.H.); hailongzyolo@163.com (H.Z.); 19122968567@163.com (M.M.); 2 Key Laboratory of Cancer Prevention and Treatment of Guizhou Province, Zunyi 563000, China; 3 Collaborative Innovation Center of Tissue Damage Repair and Regeneration Medicine, Zunyi Medical University, Zunyi 563000, China; 4 Guizhou Biomanufacturing Laboratory, Zunyi Medical University, Zunyi, 563000, China

**Keywords:** plant-derived exosome-like nanovesicles, microneedle, transdermal delivery, skin disease, psoriasis, atopic dermatitis, wound healing, cosmeceutical, extracellular vesicles, nanomedicine

## Abstract

Plant-derived extracellular vesicle-like nanovesicles (PELNs) are being investigated as plant-derived preparations with intrinsic bioactivity and potential utility as nanocarriers. Microneedles (MNs) can create transient skin microchannels and may support localized intradermal delivery of vesicle-containing formulations. The intersection of these approaches is promising, but the available evidence remains heterogeneous and includes direct PELN–MN studies, PELN studies without MNs, and related mammalian extracellular vesicle platforms. Here we provide a comprehensive review of PELN identity, isolation, characterization, cargo, and proposed dermatological activities, followed by an assessment of MN designs relevant to vesicle delivery. We searched PubMed, Web of Science, Scopus, and Embase, screened records in duplicate, and classified each included study by the directness of evidence into studies providing direct PELN-MN skin evidence, PELN evidence without MNs, mammalian extracellular vesicle MN analogue evidence, and non-dermatological or conceptual evidence. Current limitations include uncertain vesicle identity, co-isolated plant constituents, variable preparation and dosing methods, limited controls for the mechanical effects of microneedling, and incomplete clinical follow-up. Progress toward translation will require source-specific quality attributes, orthogonal potency assays, transparent comparators, and adequately controlled clinical studies.

## 1. Introduction

Skin diseases affect an estimated 30–70% of the global population, imposing substantial burdens on healthcare systems and quality of life [1,2]. Inflammatory dermatoses such as psoriasis and atopic dermatitis (AD) arise from complex interactions among genetic predisposition, environmental triggers, immune dysregulation, and epidermal barrier dysfunction [3,4]. Chronic wounds, hypertrophic scarring, photoaging, and pigmentary disorders further expand the clinical spectrum of unmet dermatological needs [5,6]. Conventional therapies—topical corticosteroids, calcineurin inhibitors, systemic immunosuppressants, and biologic agents—are frequently constrained by inadequate skin penetration, off-target effects, systemic toxicity, and high treatment costs [7,8]. These limitations underscore the urgent need for innovative therapeutic platforms that integrate targeted delivery, favorable safety profiles, and multi-mechanistic efficacy.

Over the past decade, extracellular vesicles (EVs), particularly exosomes, have attracted intense interest as next-generation therapeutic agents and drug delivery vehicles [9,10]. While mammalian cell-derived exosomes—especially those from mesenchymal stem cells (MSCs)—have dominated the field, their clinical translation faces persistent challenges, including low production yields, batch-to-batch heterogeneity, potential oncogenic risks, and complex regulatory pathways associated with living cell-based products [11,12]. These limitations have catalyzed growing interest in plant-derived exosome-like nanovesicles (PELNs), also referred to as plant-derived nanovesicles (PDNVs), plant-derived extracellular vesicles (PDEVs), or exosome-like nanoparticles (ELNs) [13,14].

PELN is used here as an operational term for plant-derived, vesicle-enriched nanovesicle preparations, typically reported in the 30–300 nm range; their cellular origin and composition vary with source and isolation method [15,16]. Following MISEV2023 and current plant extracellular vesicle guidance [17,18], we restrict the term “extracellular vesicle” to particles whose endosomal or plasma-membrane origin has been experimentally demonstrated. Preparations obtained from homogenized tissues, juices, or commercial callus-derived “stem-cell exosome” products are termed vesicle-enriched preparations, because co-isolated protein, polysaccharide, and phytochemical components cannot be excluded and exosomal biogenesis is not established. We use the nomenclature of each cited study when reporting its findings, but we do not infer exosomal identity beyond what that study demonstrates. They carry a rich and complex cargo of lipids, proteins, nucleic acids (particularly microRNAs), and plant-specific secondary metabolites such as polyphenols, and flavonoids [19]. Potential advantages include broad plant sourcing, intrinsic constituents, and the possibility of combining carrier and bioactive functions. These advantages remain dependent on source material, purification, dose, and preparation quality. Reports of plant miRNA or metabolite activity in mammalian systems do not by themselves establish cross-kingdom therapeutic activity in skin [20,21,22]. Preclinical studies and small early human reports have suggested potential activity of selected plant-derived vesicle preparations across several skin-related settings, including wound healing, skin cancer, inflammatory dermatoses, photoaging, and pigmentary disorders [13,23,24,25].

However, a fundamental bottleneck limiting the dermatological application of PELNs—and indeed all topically applied nanotherapeutics—is the formidable barrier posed by the stratum corneum, the outermost layer of the epidermis [26,27]. Intact skin restricts the passive penetration of nanoparticles larger than approximately 10–20 nm, effectively excluding the vast majority of PELNs (typically 50–300 nm in diameter) from reaching viable epidermal and dermal target cells [28,29]. While passive topical application and intradermal injection have been explored, neither approach optimally balances patient convenience, delivery efficiency, and localized bioavailability [30].

Microneedle (MN) technology has emerged as a transformative solution to this delivery challenge [31,32]. MNs are micron-scale needle arrays (typically 50–1500 μm in height) that physically breach the stratum corneum to create transient microchannels, enabling direct delivery of therapeutic cargo—including nanoparticles, nucleic acids, and proteins—into the viable epidermis and upper dermis [33,34]. MNs combine the delivery efficiency of hypodermic injection with the convenience and minimal invasiveness of topical application, offering painless administration, reduced infection risk, and potential for self-administration [35,36]. Various MN formats have been developed for exosome delivery, including dissolvable MNs that release cargo upon dissolution in the skin, coated MNs with exosome-loaded surface layers, hydrogel-forming MNs that swell to form aqueous conduits, and hollow MNs functioning as micro-syringes [37,38,39]. Recent innovations in stimuli-responsive and wearable MN systems further expand the design space for precision transdermal delivery [40,41].

The integration of PELNs with MN delivery platforms represents a promising therapeutic concept that has received rapidly increasing research attention since 2024 [42,43]. By combining the reported bioactivity and potential safety advantages of plant-derived nanovesicles with the localized intradermal delivery enabled by MN technology, PELN-MN systems offer the potential to address key limitations of both modalities independently [44,45]. Recent studies have provided heterogeneous evidence across related settings, including Coptis chinensis-derived nanovesicle-loaded MNs for infected wound healing, ginseng exosome-loaded MN rollers for skin photoaging, Portulaca oleracea-derived vesicle-loaded MNs for atopic dermatitis, and rose stem cell-derived exosomes combined with clinical microneedling for scar management [43,46,47,48]. However, despite this accelerating momentum, the evidence base has not yet been critically synthesized with explicit separation of direct and indirect evidence.

We searched PubMed, Web of Science, Scopus, and Embase using a combination of the search terms: “plant-derived exosome-like nanovesicles”, “plant-derived extracellular vesicles”, “exosome-like nanoparticles”, “microneedles”, “microneedling”, “transdermal delivery”, and “skin diseases”. Reference lists of retrieved reviews were screened manually, and the search was restricted to English-language, peer-reviewed publications. Titles, abstracts, and then full texts were screened independently by two authors, with disagreements resolved by discussion. Studies were eligible if they reported plant-derived vesicle preparations, MN-mediated delivery, or their combination in a dermatological context. We excluded conference abstracts without full data, studies reporting only crude plant extracts with no vesicle isolation or characterization, and non-cutaneous studies unless cited explicitly as platform analogues. Because the field is small, methodologically heterogeneous, and dominated by early-stage reports, we instead graded every included study by the directness of evidence: Tier D1, direct plant-derived vesicle–MN evidence in skin; Tier D2, plant-derived vesicle evidence without MN delivery; Tier D3, evidence for mammalian extracellular vesicle–MN analogues; and Tier D4, non-dermatological or conceptual extrapolation. We applied this grading to every entry in Table 1 and reported the results.

**Figure 1:**
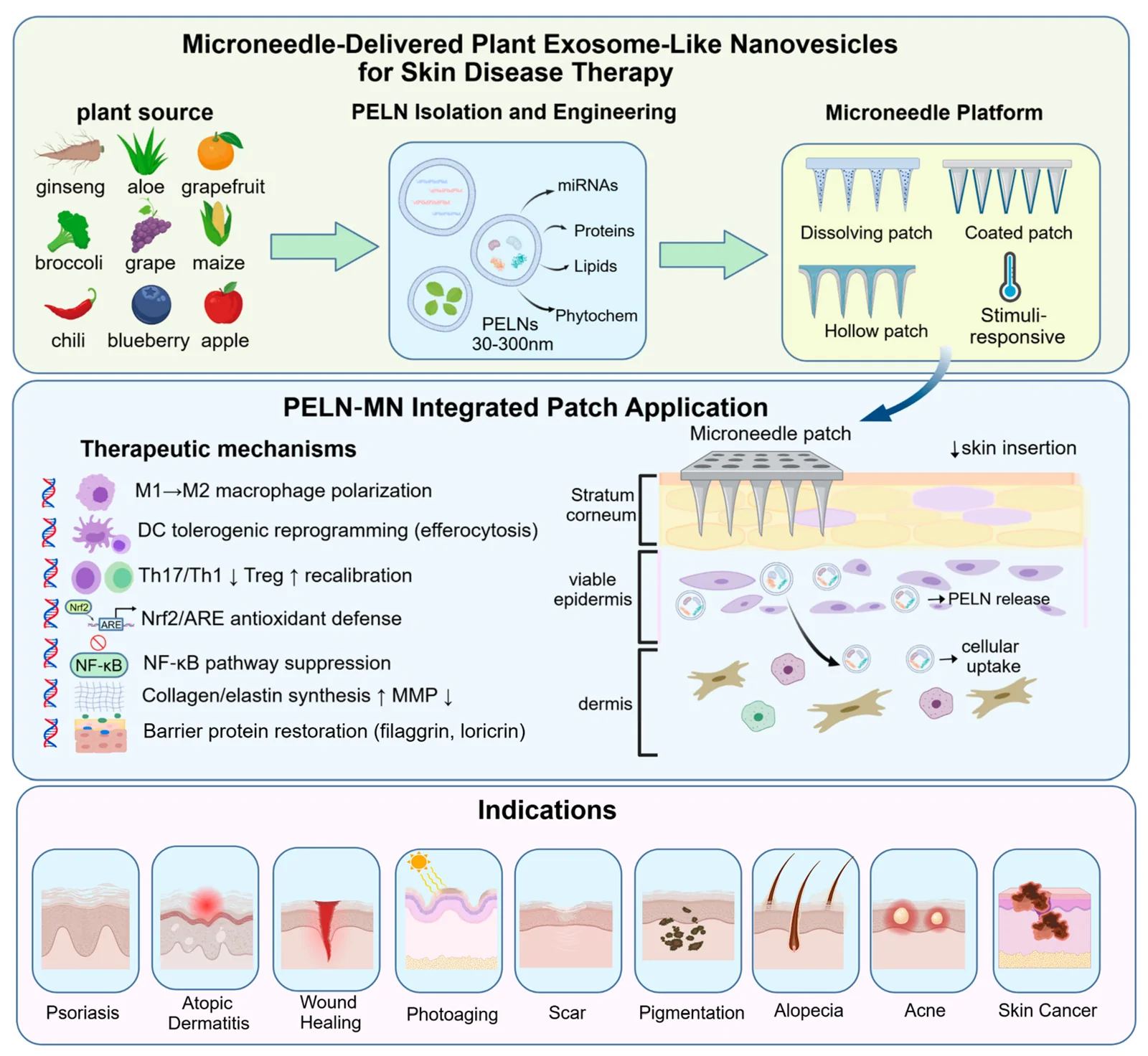
Schematic overview of the integration of plant-derived exosome-like nanovesicles (PELNs) with microneedle (MN) technology for dermatological applications.Created with BioGDP.com [49].

This review examines PELN-containing formulations and MN-mediated skin delivery. We summarize the operational definition, proposed biogenesis, isolation, characterization, and engineering of PELNs, then synthesize their reported cargo and dermatological activities while distinguishing source- and model-specific observations from untested mechanisms. We further review MN platforms and separate direct PELN-MN evidence from PELN-only, mammalian-EV, non-dermatological, and conceptual evidence. Finally, we assess the limited clinical literature and outline priorities for standardization, manufacturing, regulation, and controlled clinical evaluation. The conceptual integration of plant-derived vesicle preparations and MN delivery is illustrated in Figure 1.

## 2. Plant-Derived Exosome-like Nanovesicles: Biogenesis, Isolation, and Characterization

### 2.1. Biogenesis and Structural Features

Plant cells produce extracellular vesicles through evolutionarily conserved mechanisms that share fundamental similarities with mammalian exosome biogenesis, yet exhibit plant-specific features [15,16]. Three principal pathways contribute to PELN formation.

The multivesicular body (MVB) pathway has been proposed as one route contributing to plant extracellular vesicle-like preparations. Late endosomes/MVBs containing intraluminal vesicles (ILVs) fuse with the plasma membrane, releasing their ILV cargo into the apoplast as exosome-like nanovesicles [50,51]. This process involves the Endosomal Sorting Complex Required for Transport (ESCRT) machinery, tetraspanin proteins (notably TET8 and TET9 in plants), and Rab GTPases [52,53]. The relative contribution of this route to operationally isolated PELN preparations remains source- and method-dependent.

The exocyst-positive organelle (EXPO) pathway has been described as a plant unconventional secretion route. EXPOs are double-membrane organelles that fuse with the plasma membrane to release single-membrane vesicles into the extracellular space, independent of the Golgi apparatus and MVBs [54,55]. EXPO-associated vesicles may differ in size and cargo from other plant vesicle populations; their inclusion within operational PELN preparations requires source-specific verification.

A further proposed route involves vacuole–plasma membrane fusion, wherein the central vacuole—a defining organelle of plant cells—directly fuses with the plasma membrane to release its contents, including vesicular structures, into the apoplast [23,56]. Whether this pathway contributes materially to isolated PELN populations remains unresolved.

The structural organization of PELNs can resemble that of mammalian extracellular vesicles. They are composed of a phospholipid bilayer membrane enclosing an aqueous lumen, with typical diameters ranging from 30 to 300 nm, although size distributions vary considerably depending on the plant source, isolation method, and subpopulation analyzed [20,57]. The lipid composition of PELN membranes is distinctive: in addition to phosphatidylcholine (PC) and phosphatidylethanolamine (PE) common to mammalian EVs, PELNs are enriched in plant-specific lipids including phosphatidic acid (PA), monogalactosyldiacylglycerol (MGDG), digalactosyldiacylglycerol (DGDG), and phytosterols [58,59]. The relative abundance of these lipid species varies markedly between plant sources—for instance, ginger-derived PELNs contain high levels of PA (~25–40% of total lipids), whereas grapefruit-derived nanovesicles are enriched in PC [20,60]. These lipid compositions influence PELN stability, cellular uptake mechanisms, and tissue biodistribution.

### 2.2. Isolation and Purification Methods

The isolation of high-purity, functionally intact PELNs from complex plant matrices represents a critical—and as yet unstandardized—challenge in the field [61,62]. Plant tissues contain abundant interfering substances, including polysaccharides (cellulose, pectin), polyphenols, pigments, and fibers, which can co-purify with PELNs and confound downstream analyses.

Differential ultracentrifugation (dUC) remains the most widely employed isolation method. The typical workflow involves low-speed centrifugation steps (400–1000× *g*) to remove cells, debris, and large organelles, followed by intermediate-speed spins (10,000–20,000× *g*) to pellet larger vesicles and aggregates, and finally high-speed ultracentrifugation (100,000–150,000× *g*) to sediment PELNs [17,63]. While dUC produces PELN preparations of acceptable purity for many applications, it has notable limitations, including co-sedimentation of non-vesicular contaminants (for example, protein aggregates, lipoprotein particles, polysaccharide complexes), potential vesicle damage from high gravitational forces, batch-to-batch variability, and limited scalability.

Density gradient centrifugation (typically using sucrose or iodixanol/OptiPrep gradients) provides superior purity by separating vesicles based on buoyant density [25,64]. This method effectively removes many non-vesicular contaminants and can resolve PELN subpopulations, but is labor-intensive, time-consuming, and low-throughput.

Size-exclusion chromatography (SEC) can be chosen as a standard method for plant nanovesicle isolation, preserving functionality [65,66,67,68]. SEC columns packed with cross-linked agarose or Sepharose resins allow PELNs to elute in the void volume while retaining smaller soluble proteins and metabolites. SEC is increasingly recommended as a complementary or alternative method to dUC, particularly for functional studies.

Polymer-based precipitation (for example, polyethylene glycol/PEG) offers high yields and simplicity but at the cost of lower purity due to co-precipitation of non-vesicular macromolecules [67,68]. Commercial kits adapted from mammalian exosome isolation have been applied to plant samples with variable success.

Emerging technologies include tangential flow filtration (TFF), which enables scalable processing compatible with Good Manufacturing Practice (GMP) with good yield and purity; microfluidic-based isolation, offering high-resolution separation with minimal sample volumes; and immunoaffinity capture targeting putative plant EV surface markers, though the lack of universally accepted plant EV markers currently limits this approach [67,68].

A growing consensus in the field advocates for combinatorial isolation strategies—for example, dUC followed by SEC or density gradient centrifugation—to balance yield, purity, and functional integrity [67,68]. The choice of isolation method must be carefully matched to the intended application, as therapeutic-grade PELNs for clinical use will likely require more rigorous purification than those intended for mechanistic research.

### 2.3. Characterization and Quality Control

Comprehensive characterization of PELN preparations is essential for reproducibility, comparability across studies, and eventual regulatory approval. The International Society for Extracellular Vesicles (ISEV) has published consensus guidelines (MISEV2018, updated as MISEV2023) that, while primarily developed for mammalian EVs, provide a useful framework for PELN characterization [18,69].

Physical characterization covers particle size, concentration, morphology, and surface charge. Particle size distribution and concentration are measured by nanoparticle tracking analysis (NTA), dynamic light scattering (DLS), tunable resistive pulse sensing (TRPS/RPS), and electron microscopy-based methods. Morphology is assessed by transmission electron microscopy (TEM) with negative staining, which reveals the characteristic cup-shaped appearance of desiccated vesicles, whereas cryo-electron microscopy (cryo-EM) preserves native spherical morphology and is increasingly preferred. Surface charge, quantified by zeta potential, reflects colloidal stability and may influence skin penetration and cellular uptake.

Biochemical characterization encompasses protein, lipid, and nucleic acid content. Protein content is quantified by BCA or Bradford assays, proteomic profiling is performed by mass spectrometry, and putative plant EV markers such as PEN1, PATL1, heat shock proteins HSP70/HSP90, aquaporins, and metabolic enzymes are detected; however, universally accepted plant EV-specific protein markers analogous to the mammalian EV tetraspanins CD9, CD63, and CD81 are still lacking [16,70]. Lipidomic analysis reveals plant-specific glycerolipids (MGDG, DGDG, PA), phospholipids, and phytosterols that may serve as distinctive compositional signatures, whereas small RNA sequencing (sRNA-seq) profiles the plant miRNAs and other small non-coding RNAs carried by PELNs.

Functional characterization should assess cellular uptake efficiency and mechanisms such as endocytic pathways and membrane fusion; bioactivity in relevant in vitro assays, including anti-inflammatory cytokine modulation, antioxidant capacity, and wound-healing scratch assays; and stability under storage, encompassing temperature, freeze–thaw cycles, and lyophilization.

The development of standardized, fit-for-purpose characterization panels—tailored to the specific plant source, isolation method, and intended application—represents a high-priority need for advancing the field toward clinical translation [20,32,71].

## 3. Bioactive Cargo and Therapeutic Mechanisms in Skin Disease

**Figure 2:**
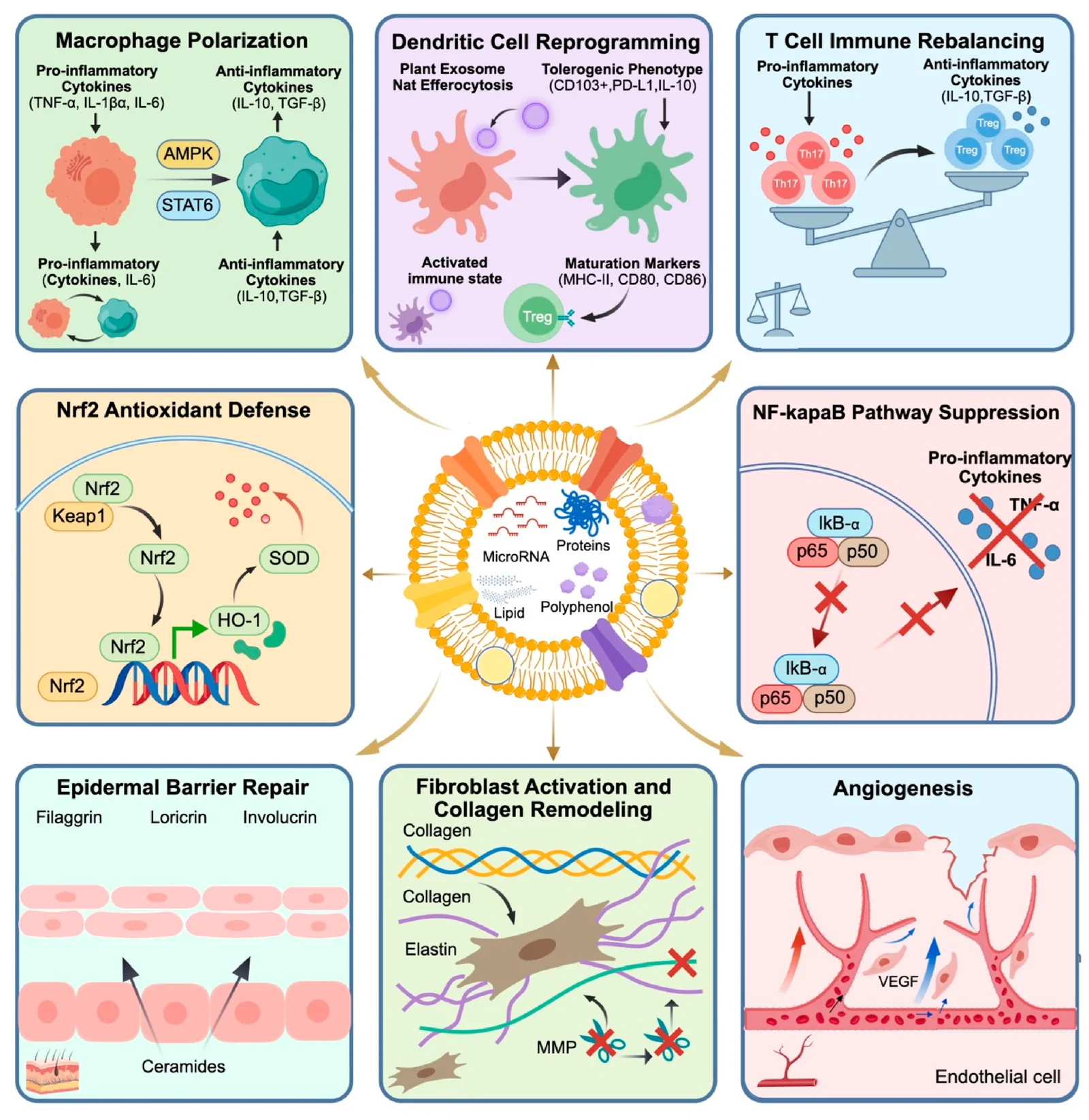
Multicomponent cargo and proposed therapeutic mechanisms of plant-derived vesicle preparations in skin disease.Created with BioGDP.com [49].

The following sections distinguish composition, observed biological responses, and proposed mechanisms. Because plant sources, isolation procedures, and disease models vary substantially, mechanistic interpretations are reported as preparation- and model-specific rather than as universal properties of PELNs. Figure 2 summarizes these cargo-related mechanistic relationships.

### 3.1. Lipid, Protein, and Nucleic Acid Composition

Reported dermatological activities of PELN preparations may reflect their multicomponent cargo, although the relative contributions of intact vesicles, co-isolated constituents, and individual cargo classes remain difficult to resolve [72].

PELN membranes are enriched in bioactive lipids that contribute to both structural integrity and therapeutic activity [20]. Galactolipids (MGDG, DGDG), characteristic of chloroplast membranes and abundant in green-tissue-derived PELNs, possess intrinsic anti-inflammatory properties and may act as peroxisome proliferator-activated receptor (PPAR) ligands [73,74]. Phytosterols contribute to membrane stability and have cholesterol-lowering and anti-inflammatory activities relevant to skin health. The distinctive lipid composition of PELNs also influences their interaction with skin lipids and their ability to penetrate or fuse with keratinocyte membranes [13,75].

Proteomic analyses have identified hundreds to thousands of proteins in PELN preparations, including metabolic enzymes (GAPDH, enolase), chaperones (HSP70, HSP90), aquaporins, cytoskeletal proteins (actin, tubulin), and proteins involved in vesicle trafficking and membrane fusion (annexins, Rab GTPases, SNAREs) [76,77]. Many of these proteins may contribute to PELN bioactivity. HSP70 has immunomodulatory properties, aquaporins may facilitate hydration-dependent processes in skin, and metabolic enzymes carried by PELNs may retain catalytic activity upon cellular uptake. Defense-related proteins, including pathogenesis-related (PR) proteins, antimicrobial peptides, and protease inhibitors, are prominent in PELNs from certain plant sources and may contribute to antimicrobial and tissue-protective effects [78,79,80,81].

The small RNA repertoire of PELNs, dominated by microRNAs (miRNAs), is a well-studied class of PELN-associated cargo [77]. Some studies have reported plant miRNA-associated effects in mammalian systems, although the extent, reproducibility, and contribution of intact vesicles remain debated. Specific plant miRNAs detected in plant-derived preparations have been reported to associate with changes in mammalian transcripts in non-cutaneous models (such as miR159 targeting TCF7 in the Wnt pathway and miR168a targeting LDLRAP1) [82,83]. The miRNA cargo profiles vary substantially between plant species, tissues, and growth conditions, offering opportunities for source selection based on desired therapeutic miRNA signatures.

Phytochemicals are a defining feature that distinguishes PELNs from mammalian EVs, as they are enriched in plant-specific secondary metabolites with established dermatological bioactivity [84]. Polyphenols and flavonoids, including curcuminoids from turmeric, quercetin, resveratrol, and anthocyanins, provide antioxidant and anti-inflammatory activities by scavenging reactive oxygen species (ROS), inhibiting NF-κB and MAPK signaling, and suppressing COX-2 and iNOS expression [85]. Ginsenosides from Panax ginseng and Panax notoginseng promote angiogenesis [86], collagen synthesis, and fibroblast proliferation, whereas glycyrrhizic acid from licorice has anti-inflammatory properties [87,88]. Berberine and related alkaloids from Coptis chinensis exhibit broad-spectrum antimicrobial activity and immunomodulatory effects [89]. 6-gingerol and 6-shogaol from ginger, menthol, and limonene contribute anti-inflammatory, antimicrobial, and penetration-enhancing effects [90,91,92].

This multi-component cargo endows PELNs with a dual therapeutic-delivery functionality: they act simultaneously as intrinsic bioactive agents (through their endogenous cargo) and as natural nanocarriers for exogenous therapeutic payloads.

### 3.2. Immunomodulatory Mechanisms

Immune dysregulation is central to the pathogenesis of many skin diseases, including psoriasis (Th17/IL-23-dominant autoimmunity), AD (Th2-skewed inflammation with epidermal barrier impairment), and chronic wounds (sustained pro-inflammatory macrophage polarization). PELNs exert multi-level immunomodulatory effects that collectively promote resolution of inflammation and restoration of immune homeostasis [70,93,94].

In selected source- and model-specific studies, plant-derived vesicle preparations have been associated with a shift in macrophage polarization from M1-like toward M2-like phenotypes [43,45,95,96]. These phenotype shifts have been associated with changes in pathways, including AMPK/mTOR signaling, STAT6 activation, and NLRP3 inflammasome suppression. In the context of chronic wounds, M2-polarized macrophages promote angiogenesis, fibroblast activation, and extracellular matrix (ECM) remodeling—key processes for wound closure and tissue regeneration.

Dendritic cell (DC) modulation has been demonstrated by the pioneering work of Ma et al. using keratinocyte-derived apoptotic nanovesicles, in which phosphatidylserine (PS)-exposing nanovesicles are selectively recognized and engulfed by cutaneous dendritic cells through PS receptor-mediated efferocytosis [97]. This recognition triggers tolerogenic DC programming, characterized by reduced expression of co-stimulatory molecules (CD80, CD86), increased production of anti-inflammatory cytokines (IL-10, TGF-β), and enhanced capacity to induce regulatory T cells (Tregs). While this mechanism was originally demonstrated with mammalian apoptotic vesicles, it raises the intriguing possibility that PELNs—which also expose PS on their outer membrane leaflet—may engage similar efferocytic recognition pathways to modulate DC function.

PELN and PELN-MN systems have been shown to recalibrate pathogenic T helper cell imbalances in a disease-context-appropriate manner. In psoriasis models, EV-loaded MNs suppress Th1 and Th17 responses while promoting Treg expansion [47,98]. In AD models, PELN treatment attenuates Th2 cytokines (IL-4 and IL-13)—key drivers of the atopic inflammatory cascade [47,99]. This context-dependent immunomodulation, wherein PELNs restore immune balance rather than simply suppressing all immune activity, represents a key therapeutic advantage over broad-spectrum immunosuppressants.

Across selected PELN sources and disease models, studies have reported changes in pro-inflammatory cytokines (TNF-α, IL-1β, IL-6, IL-17, IL-23) and chemokines (CCL20, CXCL1, and CXCL8/IL-8), with variable changes in anti-inflammatory mediators [100,101]. This cytokine network reprogramming is achieved through modulation of key transcription factors, most notably NF-κB (suppressed via IκBα stabilization and inhibition of IKK complex activity), STAT1/STAT3 (context-dependent modulation), and AP-1.

### 3.3. Anti-Inflammatory and Antioxidant Activities

Oxidative stress and chronic inflammation are intimately linked in skin pathophysiology, forming self-amplifying feedback loops that perpetuate tissue damage and impair repair. PELNs are particularly well-suited to interrupt these pathogenic cycles due to their enrichment in both antioxidant phytochemicals and anti-inflammatory mediators [40,84].

Some PELN-associated preparations have been reported to modulate the Nrf2/ARE pathway, the master regulator of cellular antioxidant responses. Nrf2 activation induces transcription of a battery of cytoprotective genes, including heme oxygenase-1 (HO-1), NAD(P)H:quinone oxidoreductase 1 (NQO1), glutathione S-transferases (GSTs), and superoxide dismutase (SOD) [102,103]. Aloe vera-derived nanovesicles, ginseng-derived exosomes, and turmeric-derived vesicles have been particularly well-characterized for Nrf2 pathway activation in skin cells. Direct ROS scavenging is provided by PELN-associated polyphenols, flavonoids, and carotenoids, which can neutralize superoxide anions, hydroxyl radicals, and hydrogen peroxide.

NF-κB, the central transcriptional hub for inflammatory responses, is a key target of PELN-mediated anti-inflammatory activity. PELN cargo components—including specific miRNAs, polyphenols, and bioactive lipids—suppress NF-κB activation at multiple levels, including inhibition of IκB kinase (IKK) activity, stabilization of the NF-κB inhibitor IκBα, and interference with NF-κB DNA binding [104,105]. The extent to which these observations reflect intact vesicles or co-isolated constituents, and whether they persist in vivo, remains to be established.

Emerging evidence suggests that some plant-derived vesicle preparations may modulate NLRP3 inflammasome activation, reducing caspase-1-mediated processing and secretion of IL-1β and IL-18—cytokines critically involved in autoinflammatory skin conditions [106,107]. The molecular mechanisms may involve PELN-mediated modulation of mitochondrial ROS production, potassium efflux, and lysosomal stability, all of which regulate NLRP3 inflammasome assembly.

### 3.4. Tissue Regeneration and Barrier Repair

Beyond their immunomodulatory and anti-inflammatory functions, PELNs actively promote tissue regeneration and epidermal barrier restoration—functions of particular relevance to wound healing, photoaged skin, and barrier-deficient dermatoses such as AD [14,100,108].

PELNs from ginseng have been shown to accelerate keratinocyte proliferation and migration in scratch wound assays, mediated through activation of the PI3K/Akt and ERK/MAPK signaling pathways [109]. Ginsenosides carried by ginseng-derived nanovesicles are particularly potent stimulators of keratinocyte activity, promoting re-epithelialization in both in vitro and in vivo wound models.

PELNs stimulate dermal fibroblasts to produce collagen and elastin. Concurrently, PELNs modulate the balance between matrix metalloproteinases (MMPs, particularly MMP-1, MMP-9) and their tissue inhibitors (TIMPs), favoring ECM accumulation and organized remodeling over degradation [21,110,111,112,113]. These effects are mediated through TGF-β/Smad signaling and have been documented for PELNs from ginseng and lemon [101,114].

PELNs from Dendrobium officinale, ginseng, and Chuanxiong Rhizoma promote angiogenesis, including endothelial cell proliferation, migration, and tube formation through VEGF/Akt/eNOS pathway activation [115,116,117]. Specific miRNAs enriched in these PELNs (such as miR-21-3p, implicated in VEGF pathway activation) and bioactive lipids may contribute to pro-angiogenic activity. In wound healing contexts, timely angiogenesis is essential for delivering oxygen, nutrients, and immune cells to the regenerating tissue.

Some PELN preparations have been associated with changes in epidermal barrier proteins and lipids in selected models [25]. Turmeric-derived nanovesicles, in particular, have been shown to synergistically restore both the proteinaceous (cornified envelope) and lipid (lamellar body-derived) components of the epidermal barrier in AD models. This barrier-restorative activity is complemented by antimicrobial peptide induction (hBD-2, hBD-3, LL-37), enhancing innate immune defense at the skin surface.

## 4. Microneedle Technologies for PELN Delivery

### 4.1. Microneedle Types and Fabrication Strategies

Microneedles are micron-scale projections, typically 50–1500 μm in height with tip radii of 1–25 μm, arrayed on a backing layer to create patches containing tens to thousands of needles per square centimeter [31,33]. The physical dimensions are carefully designed to penetrate the stratum corneum (10–20 μm thick) and viable epidermis (50–100 μm) while avoiding contact with dermal nerve endings and blood vessels, supporting minimally invasive local delivery.

Dissolvable MNs (DMNs) are fabricated from water-soluble, biocompatible polymers—most commonly hyaluronic acid (HA), polyvinylpyrrolidone (PVP), polyvinyl alcohol (PVA), carboxymethyl cellulose (CMC), and various sugars (maltose, trehalose, sucrose) [34,118]. PELN cargo is incorporated into the polymer matrix during MN fabrication (typically by solvent casting into polydimethylsiloxane/PDMS molds followed by desiccation). Upon skin insertion, the polymeric matrix absorbs interstitial fluid and gradually dissolves, releasing PELNs into the viable epidermis and superficial dermis. DMNs offer several advantages for PELN delivery, including the absence of biohazardous sharp waste after dissolution, tunable release kinetics through polymer selection and crosslinking density, one-step application without post-removal requirements, and biocompatible matrix materials such as HA that may provide additional therapeutic benefits such as skin hydration. The principal challenges are maintaining PELN stability during the fabrication (drying) process and achieving sufficient mechanical strength for reliable skin penetration.

Coated MNs (CMNs) comprise solid MN arrays (typically silicon, metal, or non-dissolving polymer) onto which a PELN-containing coating formulation is deposited, usually by dip-coating, spray-coating, or inkjet printing [35]. Upon skin insertion, the coating dissolves in interstitial fluid, rapidly releasing PELNs. CMNs provide high mechanical robustness and rapid delivery, but are limited by the small volume of coating that can be applied (typically nanograms to low micrograms per needle) and the requirement for sharp waste disposal after use.

Hydrogel-forming MNs (HFMNs) are fabricated from crosslinked, swellable polymers that absorb interstitial fluid upon skin insertion without dissolving [36,119]. The swollen hydrogel network forms aqueous microchannels connecting the dermal interstitial space to an external drug reservoir (often integrated into the MN patch backing), enabling sustained, rate-controlled PELN delivery over hours to days. HFMNs combine strong skin adhesion, sustained release profiles, and minimal polymer residue deposition, but require a separate drug reservoir and diffusion-driven delivery kinetics that may be less suitable for large nanoparticles.

Hollow MNs (HMNs) contain a central lumen connected to a reservoir, functioning as micron-scale syringes for pressure-driven infusion of PELN suspensions into the skin [37]. HMNs enable precisely controlled dosing volumes and rates, but face challenges including clogging by viscous or aggregated PELN preparations, more complex and costly fabrication, and the need for external pressure control systems.

Core–shell and multi-layer MNs represent advanced designs in which PELNs are selectively localized within specific MN compartments—for example, concentrated in rapidly dissolving tips or embedded in core regions protected by a mechanically robust shell [38]. These architectures enable programmed, sequential release of different PELN populations or co-delivery of PELNs with complementary therapeutics.

### 4.2. Integration of Plant-Derived Vesicles with Microneedle Platforms

The integration of PELNs into MN systems introduces several technical considerations that critically influence delivery efficiency and therapeutic efficacy [120,121]. PELNs may be mixed directly with the MN matrix polymer solution before casting, deposited onto MN surfaces after fabrication, loaded into integrated drug reservoirs for hydrogel-forming or hollow MNs, or encapsulated within nano-/microparticles that are subsequently incorporated into the matrix [122]. Direct mixing is the simplest approach but may expose PELNs to denaturing conditions such as organic solvents, elevated temperatures, and shear forces during fabrication; PELN integrity can instead be preserved through mild aqueous-based fabrication, stabilizing excipients such as trehalose, sucrose, and mannitol, and post-fabrication loading onto pre-formed MNs [123].

PELN stability within the MN matrix during fabrication, storage, and skin insertion is a critical but often under-evaluated parameter. Key stability challenges include desiccation-induced membrane damage during drying, oxidative degradation of unsaturated lipids and polyphenols, aggregation arising from polymer-induced depletion forces, and enzymatic degradation by residual plant proteases or skin-resident enzymes, although the inclusion of antioxidants such as ascorbic acid and α-tocopherol, protease inhibitors, and cryoprotectants can improve stability [124]. The release of PELNs from MN matrices is governed by polymer dissolution and swelling kinetics together with PELN-matrix interactions. Rapid dissolution matrices such as low-molecular-weight HA and PVP provide burst release within minutes of skin insertion, whereas slowly dissolving or crosslinked matrices such as high-molecular-weight HA, chitosan, and methacrylated gelatin (GelMA) enable sustained release over hours, and core–shell designs offer the greatest flexibility for programming release profiles [125]. Finally, PELN incorporation may alter mechanical properties such as Young’s modulus, fracture force, and insertion depth that are critical for reliable stratum corneum penetration, so PELN loading fraction and matrix polymer selection must be optimized to avoid compromising skin penetration [126].

### 4.3. Engineering Strategies for Enhanced Delivery

Beyond MN architecture, vesicle engineering itself is directly relevant to PELN-MN systems and is considered here as a complementary design axis. Cargo can be introduced endogenously, by treating source plants or cultured plant cells so that vesicles are secreted pre-loaded, or exogenously, by electroporation, sonication, saponin permeabilization, freeze–thaw cycling, or incubation, each of which trades loading efficiency against membrane damage. Surface and membrane modification includes covalent conjugation to membrane proteins, lipid-anchored insertion of targeting ligands, electrostatic coating, and fusion with liposomes or cell-derived membranes to create hybrid vesicles. Such modifications have been used to promote uptake by keratinocytes, fibroblasts, or immune cells and to favor cytosolic release over lysosomal degradation [39,65,127]. These strategies were developed predominantly for mammalian vesicles, and their transfer to plant-derived preparations is incompletely validated. Their integration with MN design is constrained in a specific way: engineered surface ligands and fusogenic membranes must survive the casting, drying, and insertion steps of MN fabrication, and to our knowledge no study has yet reported an engineered plant-derived vesicle whose targeting function was verified after incorporation into an MN array. We therefore treat vesicle engineering and MN fabrication as coupled rather than independent problems.

Recent innovations have expanded PELN-MN systems beyond simple transdermal delivery [128]. Stimuli-responsive MNs engineered to respond to disease-specific microenvironmental cues, including elevated ROS, decreased pH, matrix metalloproteinase (MMP) activity, and elevated local temperature in inflamed skin, could enable on-demand, site-specific release of vesicle-containing formulations. Examples include ROS-responsive thioketal-based linkers incorporated into MN matrices, pH-sensitive acetal/ketal crosslinkers that hydrolyze in the mildly acidic milieu of inflamed skin (pH 5.5–6.5), and MMP-cleavable peptide sequences that are selectively degraded in chronic wound environments characterized by excessive MMP activity.

The integration of MN arrays with wearable electronic components has been proposed as a route to feedback-controlled delivery [129], although direct PELN evidence remains limited. A pioneering example is the 3D-printed, cisplatin-loaded Taxus chinensis exosome MN patch for triple-negative breast cancer, which incorporates intelligent, cascade-targeting design principles [130]. This oncology example illustrates a design concept; its performance and relevance for chronic dermatological PELN delivery remain to be tested.

Biomimetic MN designs inspired by natural structures have been applied to improve skin adhesion, delivery uniformity, and patient comfort. The hierarchical short microneedle-cupping dual-amplified patch developed by Park and colleagues incorporates octopus sucker-inspired micro-cups between short MNs (<300 μm), generating negative pressure upon application that enhances skin adhesion and temporarily deforms the stratum corneum to facilitate deeper delivery [121,131]. This design achieved a 2.6-fold increase in delivery depth compared to conventional MN patches and maintained strong adhesion even on curved or moist skin surfaces.

PELN-MN systems are increasingly combined with complementary therapeutic modalities, although formal synergy requires dedicated interaction analyses. Such combinations include PELN-MNs with non-ablative fractional laser pre-treatment to enhance skin permeability, PELNs integrated with MSC-derived biomimetic nanovesicles to combine plant-derived phytochemical bioactivity with mammalian EV-mediated signaling, and PELNs co-loaded with small-molecule drugs or nucleic acid therapeutics (miRNA, siRNA) within MN matrices. Adaptable indwelling microneedle designs have likewise been developed to improve wound-bed retention and uniform delivery [131].

## 5. Therapeutic Applications in Skin Diseases

### 5.1. Psoriasis

Psoriasis is a chronic, immune-mediated inflammatory skin disease affecting 2–3% of the global population, characterized by well-demarcated erythematous plaques with silvery scale, driven by the IL-23/Th17 inflammatory axis with contributions from TNF-α, dendritic cells, and keratinocyte hyperproliferation [3,132]. Despite the availability of highly effective biologics (anti-IL-17, anti-IL-23, anti-TNF-α monoclonal antibodies), many patients experience inadequate responses, loss of efficacy over time, adverse effects (including increased infection risk), and prohibitive treatment costs [7,133]. PELN-MN systems offer a conceptually distinct therapeutic approach based on localized, multi-target immunomodulation without systemic immunosuppression.

No study to date has evaluated a plant-derived vesicle-loaded microneedle in a psoriasis model. The evidence below is therefore presented in tiers: plant-derived vesicle evidence without MN delivery (Tier D2), followed by mammalian extracellular vesicle-MN systems (Tier D3) that define the engineering state of the art but do not constitute PELN-MN evidence.

Tier D2, plant-derived vesicles without MN delivery. Plant-derived nanovesicles have been evaluated for anti-psoriatic activity, but in all cases by routes other than microneedle delivery. Shallot- and garlic (Allium)-derived PDNVs have demonstrated IL-17 modulation and NRF2 pathway activation in preclinical psoriasis models [134]. The intrinsic antimicrobial activity of garlic-derived nanovesicles provides additional benefit, given the emerging recognition of microbial dysbiosis as a contributing factor in psoriasis pathogenesis. Grapefruit-derived exosome-like nanovesicles (GEVs) fused with CCR6-overexpressing gingiva-MSC membrane nanovesicles have shown enhanced homing to inflamed sites and therapeutic efficacy in both psoriasis and AD models [3,100,135].

Tier D3, mammalian extracellular vesicle-MN analogues. Zhou et al. reported a study employing dissolvable MNs loaded with MSC-derived biomimetic nanovesicles encapsulating PCSK9 siRNA (MN-MSCNV@siPCSK9) for psoriasis immunomodulation [98]. PCSK9, canonically a regulator of LDL receptor degradation in lipid metabolism, was recently implicated in keratinocyte hyperproliferation and immune dysregulation in psoriatic skin. The integrated platform achieved efficient transdermal siRNA delivery with nanovesicle-mediated stabilization, significantly suppressing keratinocyte proliferation and pro-inflammatory cytokine secretion in vitro. In imiquimod (IMQ)-induced psoriatic mice, MN-MSCNV@siPCSK9 alleviated epidermal hyperplasia, reduced systemic inflammation, suppressed NF-κB pathway activation, and modulated Th1, Th2, and Th17 cell subsets toward immune homeostasis. This “encapsulation–delivery–modulation” tripartite strategy establishes a translatable paradigm for nucleic acid-based precision psoriasis therapy.

**Figure 3:**
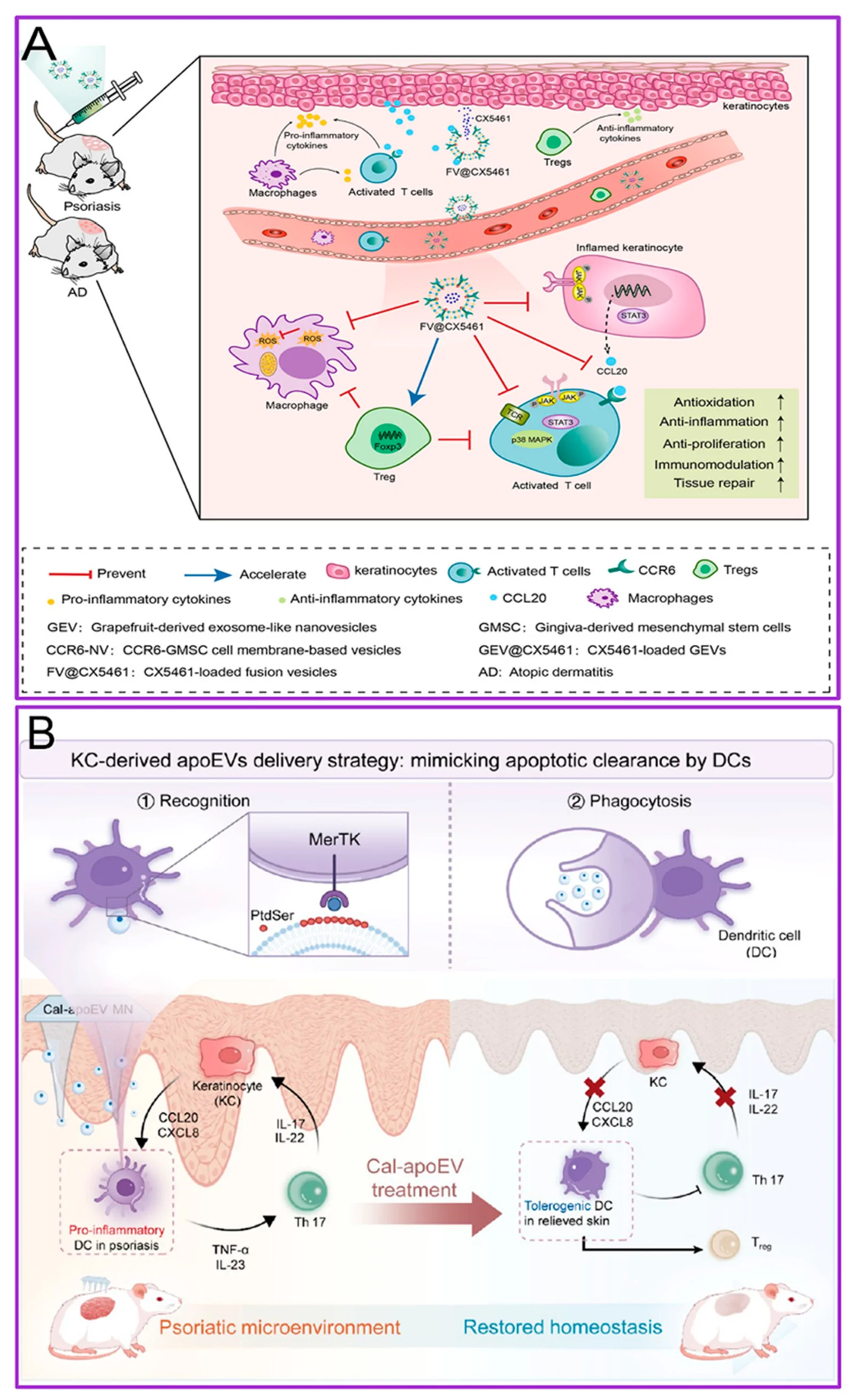
(**A**) Schematic illustration showing the preparation process of FV@CX5461 and their therapeutic effects for autoimmune skin disorders [100]. (**B**) Cal-apoEV MN-mediated targeted delivery and immunomodulation of psoriatic microenvironment. Upon skin insertion, MNs deposit Cal-apoEVs into the dermal layer. The Cal-apoEVs are subsequently internalized by DCs via surface phosphatidylserine (PtdSer) interactions, effectively reprogramming them from an immunogenic phenotype to a tolerogenic state and facilitating Treg differentiation to reestablish immune homeostasis [97].Created with BioGDP.com [49].

Also within Tier D3, Ma et al. developed a complementary approach using keratinocyte-derived apoptotic nanovesicles (apoEVs) loaded with the anti-psoriatic drug calcipotriol (Cal), integrated into dissolvable MNs (Cal-apoEV MNs) [97]. The exposed phosphatidylserine on apoEV surfaces served as an “eat-me” signal, enabling selective recognition and phagocytic engulfment by cutaneous dendritic cells through efferocytosis—the natural homeostatic process by which DCs clear apoptotic keratinocytes from the skin. This DC-specific targeting, combined with intrinsic efferocytosis-mediated immunosuppressive signaling, reprogrammed DCs from a pro-inflammatory to a tolerogenic phenotype. In the IMQ-induced psoriasis model, Cal-apoEV MNs remodeled the psoriatic immune microenvironment toward a tolerogenic state, with increased Treg infiltration and significant amelioration of psoriatic symptoms. This work elegantly exploits a natural skin homeostatic mechanism for targeted drug delivery and synergistic DC reprogramming. The two Tier D3 mammalian-vesicle systems are depicted in Figure 3.

Beyond vesicular cargo, non-vesicular MN platforms offer a useful benchmark for PELN–MN design. Zhuo et al. developed selenium-functionalized dissolvable hyaluronic acid MNs carrying shikonin-loaded tetrahedral framework nucleic acids (tFNAs), combining antioxidant selenium chemistry, the anti-proliferative phytochemical shikonin, and anti-inflammatory DNA nanostructures in an imiquimod-induced psoriasis model [136]. Although this is not a PELN system, it illustrates the broader design space of natural-product and nucleic-acid cargo delivered by MNs and provides a non-vesicular comparator against which plant-vesicle–MN constructs should be evaluated.

These studies illustrate distinct and potentially complementary strategies. They do not, however, establish a PELN-MN mechanism in psoriasis, because the plant-derived evidence lacks MN delivery and the MN-delivered evidence uses mammalian vesicles. Whether a plant-derived vesicle delivered by MN would reproduce these outcomes is untested.

### 5.2. Atopic Dermatitis

Atopic dermatitis (AD) is the most common chronic inflammatory skin disease, affecting 15–20% of children and 3–10% of adults worldwide [4,137]. AD is characterized by intense pruritus, eczematous lesions, epidermal barrier dysfunction, and Th2-dominant immune dysregulation with variable Th1, Th17, and Th22 contributions depending on disease phase and patient endotype. Current therapies—emollients, topical corticosteroids, topical calcineurin inhibitors, dupilumab (anti-IL-4Rα), and JAK inhibitors—are variably effective and carry safety concerns with long-term use.

Atopic dermatitis provides the clearest Tier D1 evidence in this review, because Portulaca oleracea-derived vesicles have been formulated into a functional MN array and tested in an animal model of AD-like inflammation. Turmeric-derived vesicle hydrogels and plant-mammalian hybrid vesicles are discussed subsequently as Tier D2 evidence, because MN delivery was not part of those studies.

PELN-MN systems could in principle address both components of AD pathology—barrier dysfunction and immune dysregulation—through their combined barrier-restorative and immunomodulatory activities.

A recently reported study described ROS-scavenging MNs loaded with Portulaca oleracea L.-derived exosomes for AD therapy [47]. Portulaca oleracea (purslane), a medicinal plant with documented anti-inflammatory properties in traditional medicine, yielded exosome-like nanovesicles that were integrated into a ROS-responsive, dissolvable MN matrix. The system achieved dual functionality, in which the MN matrix itself provided ROS-scavenging activity through 4-amino-TEMPO conjugated to hyaluronic acid (HA-TEMPO), while the released Portulaca exosomes suppressed Th2 cytokines (IL-4 and IL-13) and rebalanced Th1/Th2 immunity (reduced p-STAT6). In DNCB-induced AD-like dermatitis in BALB/c mice, the integrated MN system reduced the crusted-lesion area, epidermal hyperplasia, and mast-cell infiltration.

Turmeric-derived nanovesicles encapsulated in metal–polyphenol hydrogels have been engineered into a dual-repair strategy for AD-affected skin [138]. The Fe-HD@TDNV hydrogel synergistically restored both the proteinaceous barrier (via upregulation of filaggrin, loricrin, and involucrin) and the antimicrobial barrier (via induction of hBD-2, hBD-3, and LL-37 in keratinocytes). Whether this hydrogel formulation benefits from MN-mediated delivery remains to be established.

Plant–MSC fusion nanovesicles represent a separate hybrid strategy for autoimmune skin disorders including AD [100]. By fusing PELN membranes with engineered MSC-derived nanovesicles, the fusion vesicles combine plant-derived anti-inflammatory phytochemicals with MSC membrane-associated immunomodulatory proteins and miRNAs. Decorated with targeting ligands directed to CCL20—a chemokine highly expressed in inflamed AD skin that recruits CCR6+ Th17 cells and dendritic cells—these fusion vesicles achieve targeted delivery to inflammatory foci, suppressing Th17 activation while Treg expansion.

Additional plant sources with demonstrated anti-AD activity include Aloe vera (Nrf2/HO-1-mediated antioxidant defense and skin hydration), Peucedanum japonicum (anti-inflammatory small RNAs targeting macrophage cytokine production), and a range of ELNVs derived from Traditional Chinese Medicine herbs [80,139,140]. These TCM-derived preparations benefit from standardized cultivation and processing, well-characterized phytochemical profiles, and extensive historical clinical use of the source herbs in dermatology.

### 5.3. Wound Healing and Scar Management

Impaired wound healing—encompassing acute traumatic wounds, chronic diabetic ulcers, pressure ulcers, burn wounds, and surgical incisions—and pathological scarring (hypertrophic scars and keloids) represent major clinical challenges with limited therapeutic options [5,141]. The wound healing process involves overlapping phases of hemostasis, inflammation, proliferation (granulation tissue formation, angiogenesis, re-epithelialization), and remodeling, each of which can be targeted by PELN therapeutics.

Wound healing contains the most developed Tier D1 example, a Coptis chinensis-derived vesicle-loaded dissolvable MN evaluated against free vesicles in infected full-thickness wounds. Ginseng-derived and rose callus-derived preparations follow and are graded individually because MN delivery was used in some reports and topical application in others.

Coptis chinensis-derived nanovesicle MNs represent a direct example of an integrated plant-vesicle–MN system for wound healing [43]. Coptis chinensis (Chinese goldthread) is a TCM herb rich in berberine and related protoberberine alkaloids with documented antimicrobial and anti-inflammatory activities. The soluble MN array, fabricated from sodium alginate/gelatin crosslinked with calcium ions, was loaded with Coptis-derived nanovesicles (CDVs) and evaluated in infected full-thickness skin wounds in mice. The CDV-MN system was associated with combined antibacterial, immunomodulatory, and pro-regenerative outcomes. Its antibacterial activity arose from a CDV-triggered ROS burst inside *S. aureus*, *P. aeruginosa*, and *E. coli* that led to membrane rupture and biofilm eradication; anti-inflammatory macrophage reprogramming proceeded through M1-to-M2 polarization via the AMPK/mTOR pathway, suppressing TNF-α and IL-6 while enhancing IL-10 production; and pro-regenerative effects included enhanced angiogenesis and tissue regeneration with accelerated wound closure. Critically, MN delivery significantly outperformed topical application of free CDVs, supporting the value of localized MN delivery in that model.

Ginseng-derived exosome-MN systems have been developed for wound healing and scar reduction. Ginseng (*Panax ginseng*) and *Panax notoginseng* produce PELNs rich in ginsenosides, which promote angiogenesis (via VEGF/Akt/eNOS signaling), fibroblast proliferation and collagen synthesis, and keratinocyte migration. EGF-loaded Panax notoginseng nanovesicles have been proposed as topical alternatives to conventional biologic wound healing agents [142,143]. In clinical aesthetic practice, ginseng-derived exosomes combined with microneedling have demonstrated efficacy in reducing post-inflammatory hyperpigmentation (PIH) and improving atrophic scar appearance.

Rose stem cell-derived vesicle preparations have been evaluated in small case series for scar management [48]. Majewska et al. reported the first clinical case series evaluating RSCEs (ExoSCRT™/ASCEplus Derma Signal Kit, ExoCoBio Inc., Seoul, Republic of Korea; containing ~5 billion Rosa damascena callus-derived exosomes per vial, 95–115 nm, CD9/CD63/CD81-positive) combined with microneedling (Dermapen 4.0, 0.2–0.4 mm depth) for various scar etiologies. Across treated cases—including dog bite scars, forehead trauma scars, hot oil burn scars, and iatrogenic laser complications—objective improvements were documented using the modified Vancouver Scar Scale (mVSS) and Patient and Observer Scar Assessment Scale (POSAS), with no adverse effects. One patient receiving the preparation without microneedling also showed improvement, but this observation cannot establish intrinsic transdermal penetration or the added effect of MN-created microchannels.

The reported wound-healing outcomes may involve multiple repair-associated processes—antimicrobial defense, inflammation resolution, angiogenesis, fibroblast activation, ECM remodeling, and re-epithelialization—that would be difficult to replicate with single-agent therapeutics.

### 5.4. Skin Aging and Photoaging

Skin aging, encompassing intrinsic (chronological) aging and extrinsic photoaging driven by chronic ultraviolet (UV) radiation exposure, is characterized by wrinkle formation, loss of elasticity, irregular pigmentation, telangiectasia, and dermal atrophy due to cumulative oxidative damage, ECM degradation by UV-induced MMPs, and impaired collagen/elastin biosynthesis by senescent fibroblasts [6,144]. The cosmeceutical market for anti-aging interventions is substantial and growing, yet most topical products demonstrate limited efficacy due to poor active ingredient penetration beyond the stratum corneum.

Photoaging is the only indication with human PELN-MN data from a single-arm pilot study of a ginseng-derived vesicle preparation applied with an MN roller (Tier D1). The controlled trials cited afterwards used mammalian adipose-derived vesicles and are Tier D3 comparators, not evidence for plant-derived vesicles.

Ginseng exosome-MN systems for photoaging have been advanced to human pilot studies—a milestone in PELN clinical translation [46]. The Pro@Gin-Exo platform incorporates proanthocyanidins (Pros, potent antioxidant polyphenols) loaded into ginseng root-derived exosomes via microfluidic membrane-fusion with liposomes. Delivered through a microneedle roller, Pro@Gin-Exo was evaluated in a chronic UV-induced photoaging mouse model and a pilot human study of 30 volunteers (mean age 47 years). In the animal model, the MN-delivered system significantly suppressed wrinkle formation, restored collagen density, and increased dermal thickness. In the human study, one month of treatment produced significant improvements in skin smoothness, elasticity, hydration, wrinkle depth, pigmentation, and pore size, providing preliminary evidence that warrants controlled evaluation.

Microneedling with exosomes for skin rejuvenation has been systematically reviewed. Dhaliwal et al. (2026) evaluated 8 studies (171 patients) on microneedling plus exosome therapy in the Journal of Cosmetic Dermatology [145]. In the benchmark split-face RCT by Park et al. (2023), human adipose stem cell-derived exosomes plus microneedling produced 12.4–14.4% reductions in wrinkle roughness versus 6.6–7.1% for microneedling plus saline (*p* < 0.05), with 11.3% improvement in skin elasticity, 6.5% increase in hydration, and 9.9% reduction in pigmentation, accompanied by histological evidence of increased collagen density and elastic fiber content [146]. While this study used mammalian exosomes, it provides a comparator for future studies, not direct evidence for plant-derived vesicles; transferability of the protocol remains to be tested.

Estupinan et al. (2025) compared radiofrequency microneedling plus PRP versus microneedling plus exosomes in a split-face trial of 15 patients, reporting 37% improvement in photo-aging scores at 3 months (Griffiths scale) with histological confirmation of increased collagen I, glycosaminoglycans, and collagen remodeling [147].

Additional PELN sources with anti-aging activity include Aloe vera nanovesicles (Nrf2/HO-1 antioxidant defense; protection of dermal fibroblasts from UV-induced apoptosis); Vitis vinifera (grape) nanovesicles (ROS scavenging, MMP inhibition); and Lycium barbarum (goji) nanovesicles (antioxidant protection, fibroblast preservation, collagen synthesis promotion) [79,80,148,149].

The compelling early clinical signals for MN-delivered exosome therapy in skin aging—combined with the regulatory and manufacturing advantages of plant-derived over mammalian sources—position PELN-MN systems as particularly promising for the cosmeceutical and aesthetic dermatology market, where regulatory pathways may be more navigable than for pharmaceutical indications.

### 5.5. Other Dermatological Applications

Beyond the four major disease categories discussed above, PELN-MN systems have potential applications across a broader spectrum of dermatological conditions [150].

The applications in this section are predominantly Tier D2 and Tier D4. They are included to map the research space, and none should be read as established PELN-MN evidence.

For pigmentation disorders, ginseng-derived ELNVs combined with microneedling have shown clinical efficacy in reducing post-inflammatory hyperpigmentation (PIH), with three sessions producing measurable improvement in skin tone evenness [151]. The anti-melanogenic mechanisms involve downregulation of tyrosinase (TYR), tyrosinase-related protein-1 (TYRP-1), TRP-2, and microphthalmia-associated transcription factor (MITF) through PKC/ERK pathway modulation. Rose callus-derived vesicle products have also been reported for melasma and post-inflammatory hyperpigmentation when combined with microneedling in a small case series, although the available data are descriptive and uncontrolled [140]. For vitiligo, the immunomodulatory and melanocyte-protective properties of certain PELNs warrant investigation, though published data remain very limited.

For androgenetic alopecia, PELNs from ginseng and garlic have shown hair follicle regeneration potential. Ginseng-derived ELNVs activate the Wnt/β-catenin pathway in dermal papilla cells and hair follicle stem cells, promoting anagen entry and prolonging the anagen phase [152,153]. Early, uncontrolled reports suggest that topical ginseng-derived vesicle application may increase visible hair counts, though controlled data are lacking. Garlic-derived vesicles also demonstrated hair regeneration activity via Wnt/β-catenin activation [154]. MN-mediated delivery may support follicular targeting, given that MNs can create microchannels extending to the depth of the hair follicle bulge region (the stem cell niche), although the added value of PELN delivery has not been established.

For skin cancer, grapefruit- and other Citrus (including lemon)-derived nanovesicles have shown in-vitro antiproliferative activity against melanoma cells [155]. Taxus chinensis-derived exosomes, carrying therapeutic miRNAs and engineered with tumor-targeting ligands, represent the most advanced example of plant exosome-based cancer therapy. While the primary application has been in non-cutaneous cancers (triple-negative breast cancer), the MN delivery platform is directly translatable to accessible cutaneous malignancies, where localized, sustained intratumoral delivery could maximize therapeutic index.

For acne and acne scarring, the antimicrobial and anti-inflammatory activities of PELNs from garlic, Coptis chinensis, and tea (*Camellia sinensis*) are relevant to acne vulgaris [43,95,156]. Coptis-derived CDV-MNs with demonstrated antibacterial activity against *S. aureus*—a key contributor to inflammatory acne—combined with sebocyte-modulatory and anti-inflammatory effects represent a rational multi-target therapeutic strategy. For atrophic acne scarring, the ECM remodeling and collagen-induction activities of MN-delivered ginseng and rose exosomes have shown early promise.

For infectious skin diseases, PELNs from garlic, Coptis chinensis, and tea-derived preparations have shown antimicrobial activity against selected organisms, whereas evidence for tea-derived plant vesicles and treatment of infectious skin disease remains limited, suggesting a possible direction for further research rather than an established alternative to antibiotics [43,157].

**Table 1:** Evidence base for plant-derived vesicle–microneedle systems in dermatology, graded by evidence directness. Tier D1, a direct plant-derived vesicle–MN experiment in skin; Tier D2, plant-derived vesicles studied without MN delivery; Tier D3, mammalian extracellular vesicles delivered by MN, included as engineering comparators and not as PELN evidence; Tier D4, non-cutaneous or conceptual extrapolation.

Indication	Vesicle Source and Preparation	Microneedle (MN) System	Model/Comparator	Evidence Tier	Ref.
Psoriasis	MSC biomimetic nanovesicle + PCSK9 siRNA (mammalian)	Dissolvable MN (MN-MSCNV@siPCSK9)	IMQ mouse; control groups	D3 mammalian EV + MN; animal	[98]
Psoriasis	Keratinocyte apoptotic nanovesicle (apoEV) + calcipotriol (mammalian)	Dissolvable MN (Cal-apoEV MN)	IMQ mouse; controls	D3 mammalian EV + MN; animal	[97]
Psoriasis	Shallot/garlic PDNV (IL-17/NRF2 modulation); topical hydrogel	None (topical hydrogel)	In vitro + IMQ mouse; porcine-skin ex vivo penetration	D2 PELN, no MN	[134]
Psoriasis/AD	Grapefruit GEV fused with CCR6-engineered gingiva-MSC membrane nanovesicles (FV@CX5461)	None	Psoriasis/AD mouse; inflamed-site homing	D2 plant vesicle, no MN	[100]
Atopic dermatitis	Portulaca oleracea exosome-like vesicles	Dissolvable ROS-scavenging MN (HA-TEMPO matrix)	AD mouse; MN/vehicle controls	D1 direct PELN-MN; animal + control	[47]
Atopic dermatitis	Portulaca oleracea nanovesicles (PDNV) + Portulaca-polysaccharide HA MN	Dissolvable MN (HA matrix)	DNCB-induced AD mouse; M1/M2, NF-κB/STING; EASI score, TEWL	D1 direct PELN-MN; animal + control	[45]
Atopic dermatitis	Turmeric nanovesicles (Fe-HD@TDNV metal–polyphenol hydrogel)	Hydrogel only; no MN	AD cell and animal models	D2 PELN, no MN	[138]
Atopic dermatitis	Plant-MSC fusion nanovesicles (CCL20-targeted)	None	Autoimmune-skin mouse	D2 hybrid, no MN	[100]
Atopic dermatitis	Peucedanum japonicum ELNV	None	Contact-dermatitis mouse	D2 PELN, no MN	[139]
Infected wound	Coptis chinensis nanovesicles (CDVs); sodium-alginate MN + Ca^2+^ crosslinking	Soluble dissolvable MN	Infected full-thickness mouse; FREE-CDV comparator	D1 direct PELN-MN; animal + control	[43]
Wound healing	Panax (ginseng/*P. notoginseng*) nanovesicles, EGF-loaded; QBK-2 adhesive hydrogel	Topical/adhesive hydrogel; no MN	Animal wound models; no clinical data	D2 PELN, no MN	[142,143]
Scar	Rosa damascena callus-derived product (RSCE)	Microneedling (Dermapen 4.0, 0.2–0.4 mm); one of four cases topical only	Human case series (n = 4); mVSS/POSAS; no control arm	D1 with MN; uncontrolled human	[48]
Scar/long-term	Lyophilized Rosa damascena RSCE	Microneedling and topical; includes atrophic acne scars	Human retrospective series; within-face comparison; 7 d-10 mo follow-up	D1 with MN; uncontrolled human	[114]
Photoaging	Ginseng exosome + proanthocyanidin (Pro@Gin-Exo), microfluidic liposome fusion	MN roller	UV mouse; single-arm human pilot (n = 30, mean age 47); vehicle control	D1 direct PELN-MN; animal + pilot human	[46]
Photoaging	Lycium barbarum (goji) LB-EVs, cosmetic serum matrix	None (topical serum); two-photon SC imaging	3D skin model; split-face human trial (sensitive skin); TEWL control	D2 PELN, topical, no MN; controlled split-face human	[79]
Photoaging	Human adipose-stem-cell exosomes (mammalian)	Microneedling	Human split-face RCT (within systematic review)	D3 mammalian EV + MN; human RCT	[146]
Photoaging	Mammalian exosome vs. PRP	RF microneedling	Human split-face, investigator-blinded (n = 15)	D3 mammalian EV + MN; human	[147]
Photoaging	Mixed-plant EVs (human skin fibroblasts, in vitro)	None	In vitro human fibroblasts	D2 PELN, no MN	[148]
Post-inflammatory hyperpigmentation	Panax ginseng-derived exosomes	Microneedling therapy system (MTS) + topical	Human case report (n = 2, post-acne PIH); uncontrolled	D1 with MN; uncontrolled human (n = 2)	[151]
Androgenetic alopecia	Ginsenoside Re (single compound, not a vesicle); garlic exosomes	Topical (no MN)	In vitro human dermal-papilla cells; preclinical	D2 PELN/compound, no MN; preclinical	[152,154]
Melasma/pigmentation	Rosa damascena callus-derived RSCE	Microneedling-assisted	Human case series (n = 8, mixed indications); descriptive, no response rate	D1 with MN; uncontrolled human	[140]
Skin cancer	Grapefruit nanovesicles (A375 melanoma, in vitro); Taxus TExo + cisplatin (CDDP@RKTExo-MN)	None (grapefruit)/3D-printed dissolvable MN (Taxus, non-cutaneous TNBC)	In vitro A375; 4T1 TNBC mouse	D2 in vitro + D4 non-cutaneous	[130,155]

## 6. Clinical Translation: Progress, Challenges, and Future Perspectives

### 6.1. Current Clinical Evidence

The clinical evidence base for PELN-MN systems is nascent but expanding. The most clinically documented plant-derived product is Rosa damascena stem cell-derived exosomes (RSCEs, ExoSCRT™/ASCEplus, ExoCoBio, Korea), applied with microneedling or laser therapy; published case series report scar remodeling (71–78% mVSS reduction), melasma, post-inflammatory hyperpigmentation, atopic dermatitis, and post-procedure recovery, with no adverse effects reported in these small series [114]. Ginseng-derived exosomes have been combined with microneedling for post-inflammatory hyperpigmentation and skin rejuvenation in small pilot studies, and human data exist for the Pro@Gin-Exo anti-photoaging platform (30 subjects) [46]. Additional products incorporating plant-derived EVs or EV-mimetics are available in the cosmeceutical market, predominantly in East Asia, although rigorous published studies remain sparse.

Microneedling-plus-exosome clinical evidence is dominated by the Dhaliwal et al. (2026) systematic review, which identified 8 studies (171 patients) covering skin aging, hyperpigmentation, acne scarring, enlarged pores, and androgenetic alopecia [145]. Most available studies have used mammalian, often human MSC-derived, products rather than plant-derived preparations, and plant-derived exosome clinical evidence is dominated by case series rather than controlled trials. Reported short-term tolerability has generally been favorable, with no patient discontinuations and only transient, mild local reactions such as erythema, edema, and petechiae. Key evidence gaps include the absence of published randomized controlled trials comparing PELN-MN systems with standard-of-care therapies, follow-up beyond 6 months, and rigorous control for the mechanical effects of microneedling. Heterogeneity in exosome sources, isolation methods, characterization, dosing, MN parameters, and outcome measures precludes meta-analysis, and nearly all data derive from single-center, investigator-initiated studies with inherent biases.

Graded against the tiers used in this review, the human evidence for plant-derived vesicles delivered by microneedles consists of one single-arm pilot study of a ginseng-derived preparation applied with an MN roller and one retrospective case series of a rose callus-derived product applied with microneedling. Neither included a randomized comparator, a microneedling-only arm, or blinded outcome assessment, so neither can separate the effect of the vesicle preparation from the wound-healing response induced by microneedling itself. Every controlled trial identified in this field used mammalian, predominantly adipose- or mesenchymal-stem-cell-derived, vesicles. Commercial plant-derived products are marketed principally as cosmetic rather than medicinal preparations and are not subject to equivalent efficacy review. We therefore do not transfer efficacy estimates from mammalian-vesicle trials to PELN-MN systems, and we regard the current human evidence for plant-derived vesicle-MN therapy as preliminary.

### 6.2. Manufacturing and Regulatory Hurdles

Several manufacturing and standardization barriers separate promising preclinical and early clinical data from approved products [32,71,145]. Variability in plant genetics, growth conditions, harvest timing, and post-harvest processing introduces batch-to-batch heterogeneity in PELN composition and bioactivity, so Good Agricultural and Collection Practices (GACP) are essential for pharmaceutical-grade production. Current gold-standard isolation methods such as ultracentrifugation and density gradient centrifugation are not scalable to commercial volumes, whereas tangential flow filtration (TFF), continuous-flow ultracentrifugation, and microfluidic systems are scalable alternatives that still require validation for each plant source. GMP-compliant manufacturing facilities and quality systems for PELN production are virtually nonexistent, and quality control is further limited by the absence of universally accepted plant EV identity, purity, and potency markers; the MISEV guidelines were developed for mammalian EVs and do not address plant-specific considerations such as chlorophyll contamination, polysaccharide co-isolates, and plant-specific lipid markers [18,69]. Finally, PELN stability under ambient storage is poorly characterized, and cold-chain dependence would severely limit clinical and commercial feasibility, so lyophilization and other room-temperature stabilization strategies must be developed and validated.

A source-to-product quality framework makes these risks explicit. At the cultivation stage, pesticide and herbicide residues, heavy metals, mycotoxins, and cultivar and seasonal variation all propagate into the final preparation. At harvest and processing, microbial and fungal bioburden, endotoxin, and enzymatic degradation must be controlled, and homogenization-based protocols cannot distinguish secreted vesicles from intracellular membrane fragments. At the isolation stage, co-purified polysaccharides, polyphenols, and protein aggregates contribute to measured particle counts and to apparent bioactivity. For the finished product, sterility, allergen content, plant toxin and alkaloid limits, identity and potency assays, and demonstrated batch-to-batch reproducibility are required, and immunogenicity cannot be assumed to be absent simply because the source is botanical, since plant proteins and glycans are established allergens. None of these attributes is standardized for PELN preparations at present. Accordingly, the manufacturing and regulatory advantages sometimes attributed to plant-derived vesicles are hypothetical rather than demonstrated.

The choice of vesicle source involves explicit trade-offs. Plant-derived preparations offer high input biomass, low raw-material cost, no requirement for animal-derived culture components, and no risk of human viral carryover, but they are compositionally heterogeneous, lack validated identity markers, and have limited toxicology and immunogenicity data. Mesenchymal-stem-cell-derived vesicles have the most developed characterization and GMP experience and the largest clinical dataset, but face donor variability, restricted expansion capacity, high cost, and questions regarding residual proliferative or tumor-supportive potential. Milk-derived vesicles are inexpensive and scalable and tolerate oral delivery, yet carry milk-protein allergen risk and variability with lactation stage. Bacterial and microalgal vesicles are highly scalable but raise endotoxin and immunogenicity concerns. No vesicle source of any origin currently holds FDA or EMA marketing authorization; regulators have instead issued safety communications regarding unapproved exosome products. The regulatory pathway is therefore undefined for all sources.

Challenges specific to the vesicle-MN combination deserve separate attention, because they are not inherited from either platform alone. MN fabrication subjects vesicles to organic solvents in some casting protocols, elevated temperature during polymer melting, prolonged vacuum drying or lyophilization, shear during micromolding, and osmotic stress within concentrated polymer solutions, each of which can cause membrane fusion, aggregation, cargo leakage, or loss of activity. Loading is bounded by the small volume of an MN tip, so potency per patch depends on achieving high vesicle concentration without compromising mechanical strength. Release is governed by matrix dissolution rather than by vesicle properties, and reported release is usually inferred from a surrogate label rather than from intact, functional vesicles. Critically, few studies verify vesicle integrity after fabrication, so the delivered dose of intact vesicles is generally unknown. Whether plant-derived vesicles are better suited to this environment than mammalian vesicles is plausible but unproven: their high phytosterol and galactolipid content and their phenolic cargo could confer greater rigidity and oxidative stability during drying, yet no study has compared plant-derived and mammalian vesicles for post-fabrication integrity, retained potency, or shelf life within the same MN platform [122]. We identify this direct comparison as the single most informative experiment for justifying a plant-derived vesicle-MN product.

Regulatory ambiguity further complicates translation. PELNs do not fit neatly into existing FDA/EMA categories, and as of mid-2026 no exosome product, plant-derived or otherwise, holds FDA approval, with multiple warning letters issued to clinics marketing unapproved products. Defining the active pharmaceutical ingredient for a natural, multi-component nanoparticle preparation is also challenging, complicating potency assays, batch release criteria, and demonstration of pharmaceutical equivalence. Non-clinical safety requirements, including pharmacokinetic/biodistribution, toxicology, immunogenicity, and long-term safety data, are methodologically demanding for PELNs, and clinical trial design has yet to implement standardized outcome measures, appropriate comparators, adequate follow-up, and multi-center designs. Even with regulatory approval, demonstrating cost-effectiveness relative to established therapies will be essential for market adoption.

### 6.3. Future Directions

Building on this foundation, several strategic priorities should guide future work [32,158,159]. Fundamental science should first elucidate the molecular mechanisms of PELN skin penetration, cellular uptake, intracellular trafficking, and cargo release in relevant skin cell types, define the relative contributions of different cargo components (miRNAs, proteins, lipids, and phytochemicals) to therapeutic effects through depletion and reconstitution studies, establish plant-specific EV marker panels through multi-omics characterization, and investigate the pharmacokinetic–pharmacodynamic relationship of MN-delivered PELNs, including quantitative dermal distribution, systemic exposure, and duration of effect.

Technology development should engineer PELN surface modifications such as ligand conjugation, charge modulation, and membrane fusion for enhanced skin-cell targeting, develop stimuli-responsive MN systems that achieve autonomous, disease-adaptive release based on real-time sensing of the skin microenvironment, integrate PELN-MNs with wearable electronics and digital health platforms for closed-loop management, and create systematically characterized PELN libraries as a resource for disease-specific selection and rational combination.

Manufacturing and regulatory efforts should establish GMP-compatible, scalable PELN production workflows with integrated in-line quality monitoring, develop consensus reference standards and characterization protocols through multi-stakeholder collaboration, engage proactively with regulatory agencies to clarify classification and evidentiary requirements, and standardize MN fabrication and quality-control parameters such as needle geometry, mechanical properties, PELN loading uniformity, and release kinetics.

Clinical development should conduct adequately powered, randomized, sham-controlled trials with standardized outcome measures and extended follow-up, compare PELN-MN systems head-to-head against standard-of-care therapies, investigate combination strategies with conventional topical therapies, phototherapy, laser treatment, or systemic agents, and include diverse patient populations and skin types (Fitzpatrick skin types I–VI) to ensure generalizability.

## 7. Conclusions

The convergence of plant-derived vesicle preparations and microneedle delivery is an emerging research area that may address limitations of topical administration, but its clinical value remains unestablished. Plant-derived vesicle preparations may combine broad sourcing with multicomponent cargo, although their safety and scalability require source-specific validation. MNs can create localized skin microchannels and may improve delivery to superficial skin compartments; the magnitude and reproducibility of any PELN bioavailability gain require direct measurement. Direct PELN–MN evidence remains concentrated in a limited number of preclinical skin studies, while human evidence largely concerns heterogeneous exosome or cosmetic interventions and should not be generalized to PELN–MN efficacy.

However, the field stands at a critical juncture. The gap between promising proof-of-concept studies and regulatory-approved, commercially viable products remains substantial. Bridging this gap demands concerted interdisciplinary efforts spanning fundamental vesicle biology, biomaterials engineering, scalable GMP-compliant manufacturing, regulatory science, and rigorous clinical investigation. The most urgent priorities are establishing consensus reference standards and characterization frameworks for PELN identity, purity, and potency, clarifying regulatory classification pathways through engagement with agencies, and conducting adequately powered, randomized, sham-controlled clinical trials with standardized endpoints and extended follow-up. The increasing number of studies in this space—exemplified by the rapid succession of high-impact publications since 2024 and the emergence of clinically tested products—inspires confidence that these challenges are surmountable. If they are met, PELN-MN systems hold the potential to transform the treatment landscape for skin diseases, delivering effective, safe, accessible, and sustainable therapeutics that harness the evolved biochemical wisdom of plants through the precision of modern bioengineering.

Direct plant-derived vesicle-MN evidence in skin comprises a small number of preclinical studies, of which the Coptis chinensis wound-healing and Portulaca oleracea atopic dermatitis systems are the most complete because each compared MN delivery against a non-MN control. Human evidence is limited to one single-arm pilot study and one retrospective case series; neither controlled for the biological effect of microneedling. All controlled clinical trials in this space used mammalian vesicles. Mechanistic claims rest largely on source-specific in vitro and rodent data, and no plant-derived vesicle preparation currently has validated identity or potency assays. The conclusions of this review are therefore that MN delivery is a rational and technically feasible route for plant-derived vesicles, that it has produced encouraging preclinical signals in at least two indications, and that its therapeutic value in humans is undetermined. Three studies would change this assessment: a factorial preclinical comparison of vesicle-MN against vesicle-only, MN-only, and standard care; a direct comparison of plant-derived and mammalian vesicles for post-fabrication integrity within one MN platform; and a randomized, microneedling-controlled clinical trial with a predefined endpoint.

## Data Availability

No new data were created or analyzed in this study. Data sharing is not applicable to this article.
